# Supplementary material for: Nep1-like Proteins from Valsa mali Differentially Regulate Pathogen Virulence and Response to Abiotic Stresses
Source: J Fungi (Basel). 2021 Oct 4;7(10):830. doi: 10.3390/jof7100830 (PMC8539816; doi:10.3390/jof7100830)
Supplement: Supplementary file 1 [file jof-07-00830-s001.zip › Supplementary Information-10.2.pdf]

Supplementary Information

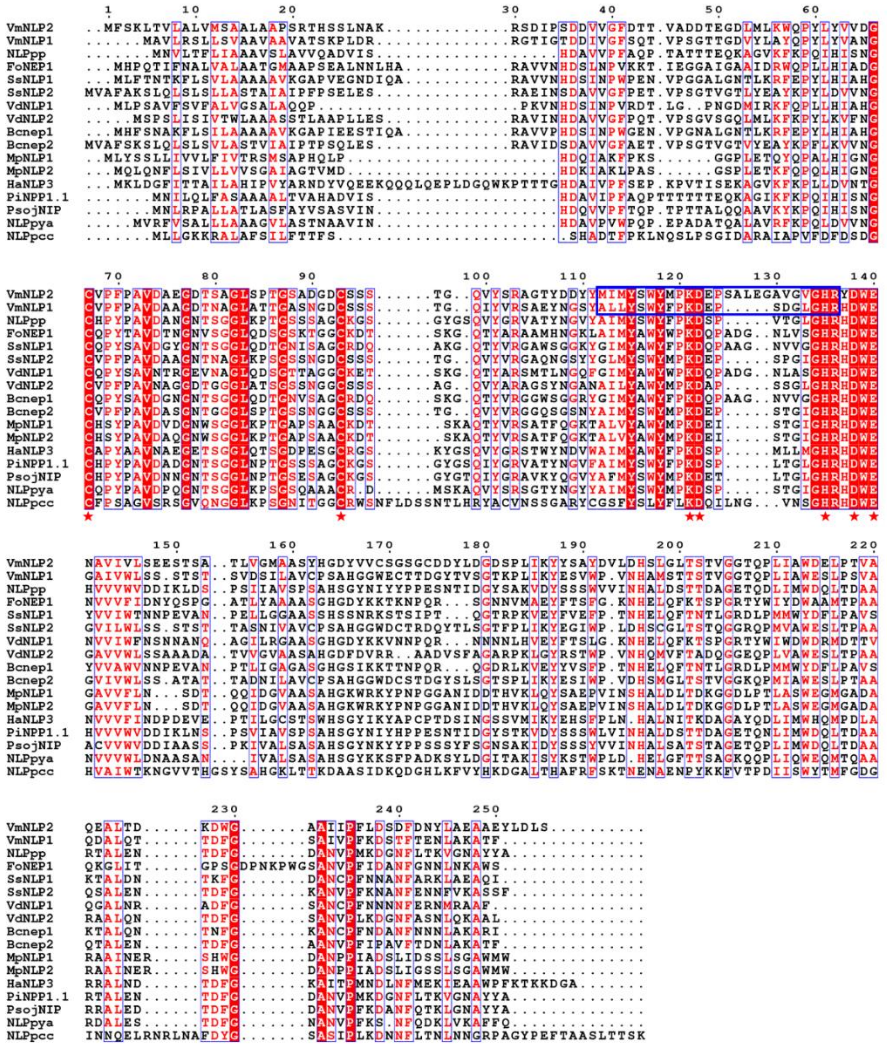

Supplementary Figure S1. Multiple sequence alignment of VmNLPs with its homologous sequences from other pathogen species using Clustal W.

VmNLP1, VmNLP2 and 15 NLPs from other phytopathogens, including NLPpp from *Parasitic phytophthora* [1], FoNep1 from *Fusarium oxysporum* [2], SsNLP1 and SsNLP2 from *Sclerotinia sclerotiorum* [3], VdNLP1 and VdNLP2 from *Verticillium dahlia* [4], Bcnep1 and Bcnep2 from *Botryis cinerea* [5], MpNLP1 and MpNLP2 from *Moniliophthora perniciosa* [6], HaNLP3 from *Hyaloperonospora arabidopsidis* [7], PiNPP1.1 from *Phytophthora infestans* [8], PsojNIP from *Phytophthora sojae* [9], NLPpya from *Pythium aphanidermatum* [10], and NLPpcc from *Pectobacterium carotovorum* [11], were selected for multiple sequence alignment by Clustal W. The bule square indicates synthesized peptides of nlp20 (VmNLP1) and nlp25 (VmNLP2) in this study. The amino acid residues that considered crucial for cytotoxic activity are indicated by red stars [12].

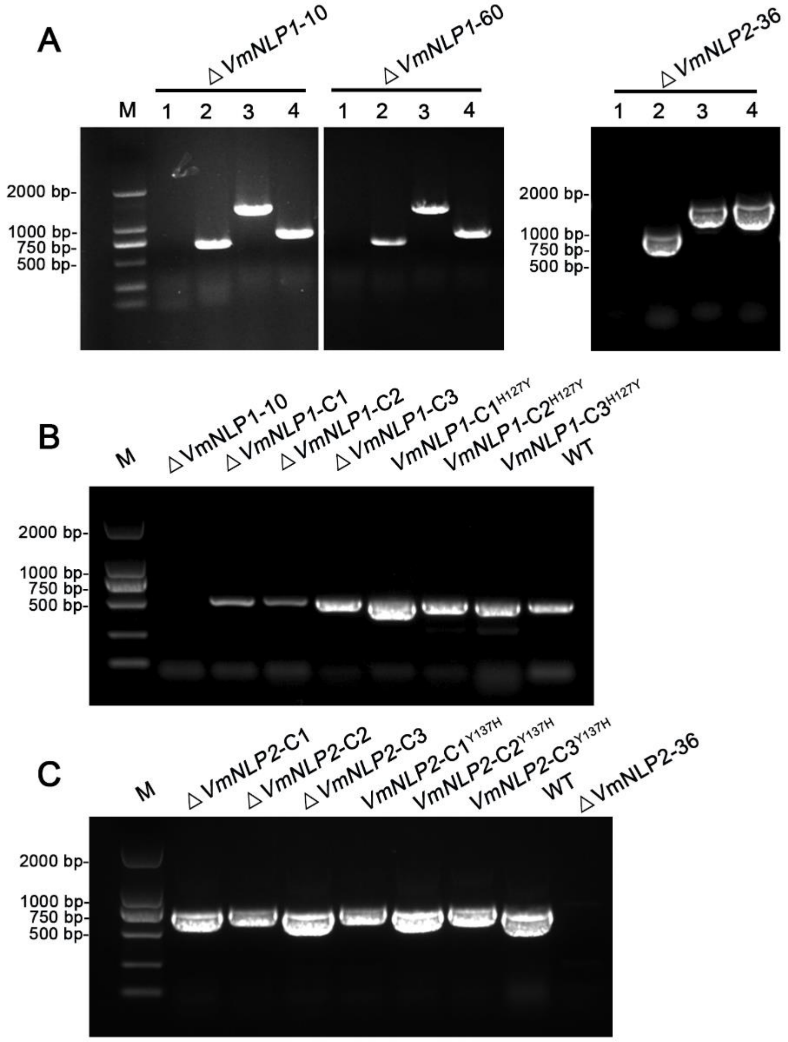

Supplementary Figure S2. Validation of gene deletion and complementation mutants.

(**A**) Two *VmNLP1* gene deletion mutants (Δ*VmNLP1*-10 and Δ*VmNLP1*-60) and one *VmNLP2* gene deletion mutants (Δ*VmNLP2*-36) were confirmed by four step Polymerase Chain Reaction (PCR) analysis: 1, targeted gene (*VmNLP1* or *VmNLP2*), detected with 5F/6R; 2, G418-resistanct cassette, detected with G852/G850; 3, upstream fusion segment, detected with 7F/GR; 4, downstream fusion segment, detected with GF/8R; M: Maker. All primer pairs were listed in Supplemental Table S1. (**B**) Validation of three different *VmNLP1* complementation transformants (Δ*VmNLP1*-C1/C2/C3), or three different complementation transformants with introduced tyrosine substitution (Δ*VmNLP1*-C1/C2/C3^{H127Y}) by PCR analysis using specific primer pairs VmNLP1-5F/6R. (**C**) Validation of three different *VmNLP2* complementation transformants (Δ*VmNLP2*-C1/C2/C3), or three different complementation transformants with introduced histidine substitution (Δ*VmNLP2*-C1/C2/C3^{Y137H}) by PCR analysis using specific primer pairs VmNLP2-5F/6R.

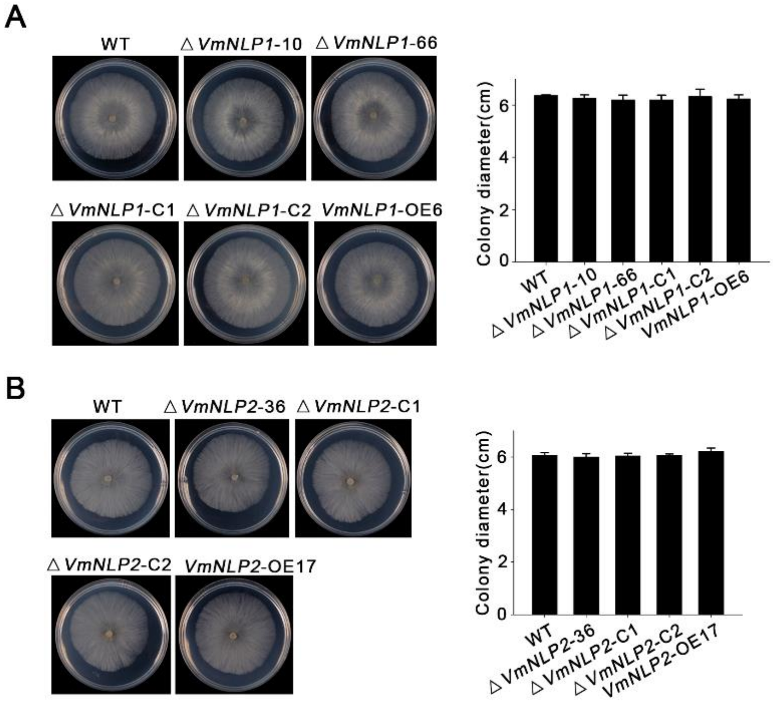

Supplementary Figure S3. Deletion of *VmNLP1* or *VmNLP2* posed no obvious influence on *V. mali* filamentous growth.

(**A**) Vegetative growth of wild type strain (WT), *VmNLP1* deletion mutants (Δ*VmNLP1*-10/66), complementation (Δ*VmNLP1*-C1/C2) and overexpression transformants (*VmNLP1*-OE6) on PDA plates. (**B**) Vegetative growth of WT, *VmNLP2* deletion (ΔVmNLP2-36), complementation (ΔVmNLP2-C1/C2) and overexpression transformants (VmNLP2-OE17) on PDA plates. All strains were maintained in the dark at 25 °C. Colony diameters were measured after cultivation for 60 hours. Bars indicate ± SD.

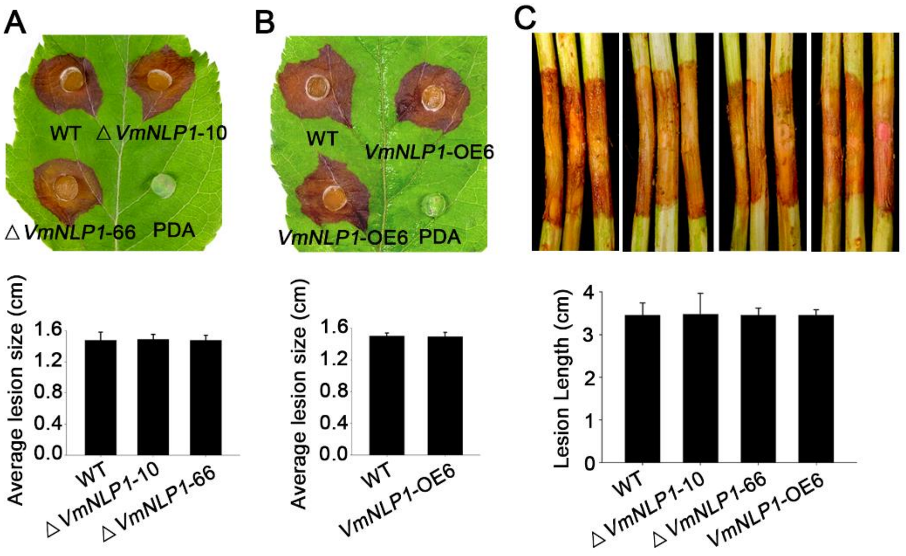

Supplementary Figure S4. Deletion of *VmNLP1* showed no apparent influence on *V. mali* virulence.

(**A**) Virulence tests of wild type (WT) and *VmNLP1* gene deletion mutants (Δ*VmNLP1*-10/66) on detached apple leaves. (**B**) Virulence tests of WT and *VmNLP1* overexpression transformant (*VmNLP1*-OE6) on detached apple leaves. Representative photographs were taken 2 d post inoculation (dpi). Average lesion sizes (lesion diameter) were measured. Bars indicate ± SD. (**C**) Virulence tests of WT, Δ*VmNLP1*-10/66 and *VmNLP1*-OE6 on detached apple twigs. Representative photographs were taken and lesion lengths were measured 4 dpi. Bars indicate ± SD.

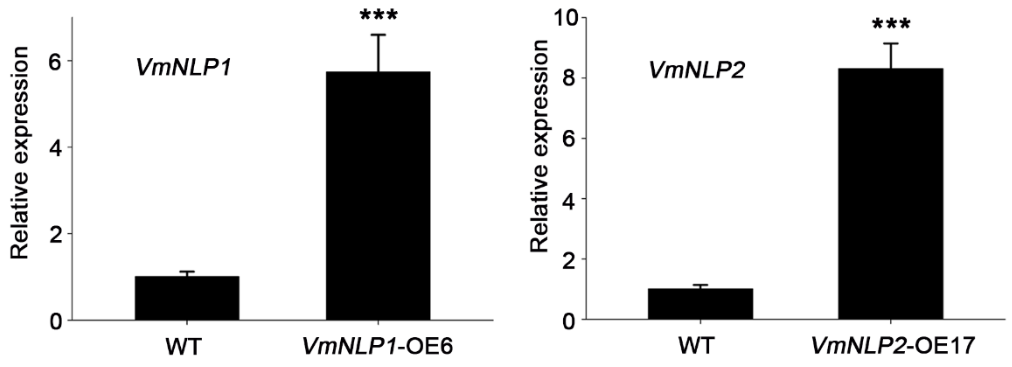

Supplementary Figure S5. Relative transcript levels of *VmNLP1* and *VmNLP2* overexpression transformants.

Hyphae samples from overexpression transformants (*VmNLP1*-OE6 and *VmNLP2*-OE17) cultured on potato PDA plates were harvested, and relative expression levels were determined by quantitative reverse transcription PCR (qRT-PCR). Transcript levels of *VmNLP1* or *VmNLP2* in wild type were set to 1, and *G6PDH* gene was used as an internal reference. Bars indicate ± SD from three technical replicates. Asterisks indicate significant differences based on Student's *t*-test (***, $P \leq 0.001$).

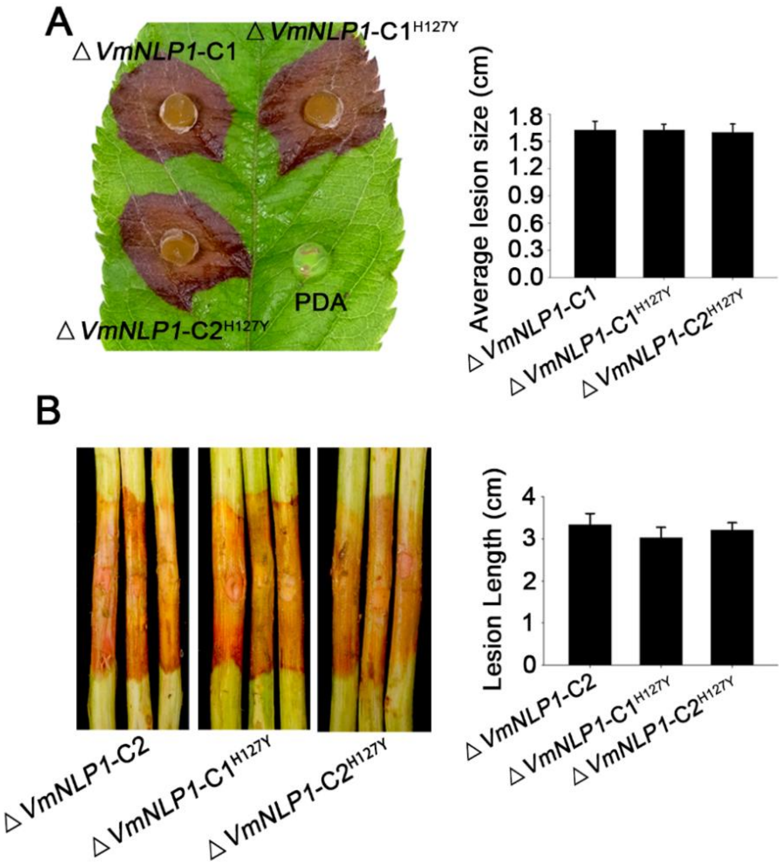

Supplementary Figure S6. Substitution of the second histidine with tyrosine in the conserved heptapeptide among VmNLP1 did not affect virulence.

Virulence tests of *VmNLP1* complementation transformant (Δ*VmNLP1*-C1) and complementation transformant with introduced tyrosine substitution (Δ*VmNLP1*-C1/C2^{H127Y}) on detached apple leaves and apple twigs. Representative photographs were taken 2 dpi for leaves and 4 dpi for twigs. Disease lesions were measured, and bars indicate ± SD.

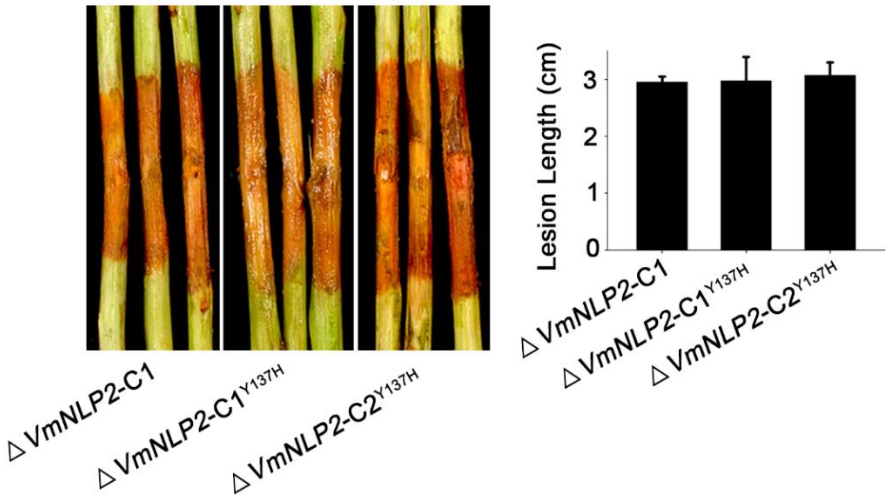

Supplementary Figure S7. Substitution of the tyrosine with histidine showed no apparent contribution to VmNLP2 virulence on apple twigs.

VmNLP2 complementation transformant (Δ*VmNLP2*-C1), and complementation transformants with introduced histidine substitution (Δ*VmNLP2*-C1/C2^{Y137H}) were inoculated on detached apple twigs. Representative photographs were taken 4 dpi. Lesion lengths were measured and bars indicate ± SD.

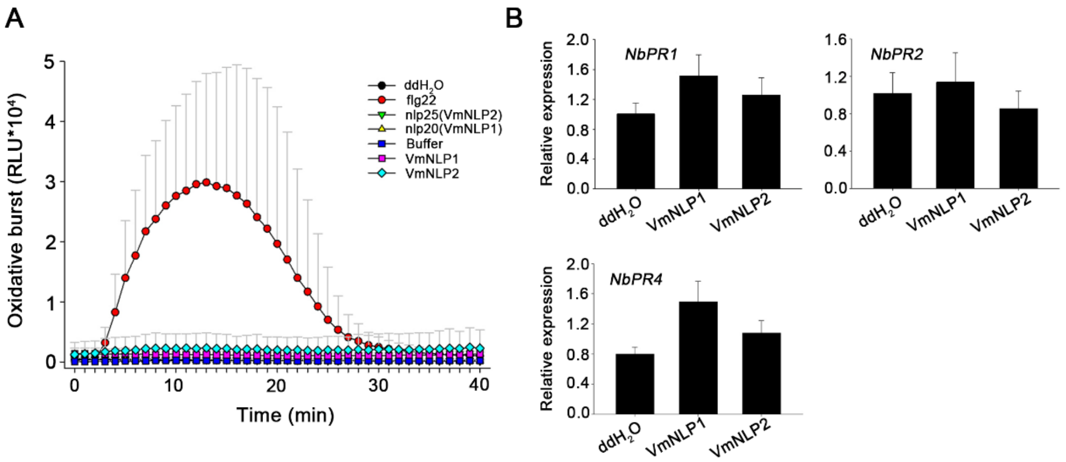

Supplementary Figure S8. VmNLP recombinant proteins and synthetic peptides are unable to trigger immune responses in *N. benthamiana* leaves.

(A) Oxidative burst in *N. benthamiana* treated with 1 μM peptides (nlp20 (VmNLP1), nlp25 (VmNLP2)) or 1 μM purified recombination proteins (VmNLP1 and VmNLP2), with ddH2O and buffer as negative controls and flg22 peptide as a positive control. Values are shown as relative light units (RLU). **(B)** VmNLP1 and VmNLP2 failed to activate obvious transcript accumulation of defense-related genes in *N. benthamiana* leaves. *N. benthamiana* leaves were infiltrated with 1 μM recombination proteins (VmNLP1 and VmNLP2) or ddH2O. Gene expression was assessed at 6 h post infiltration using qRT-PCR. *NbEF1α* was used as an endogenous reference. Means and SDs were calculated from three technical replicates. The experiments were performed three times with similar results.

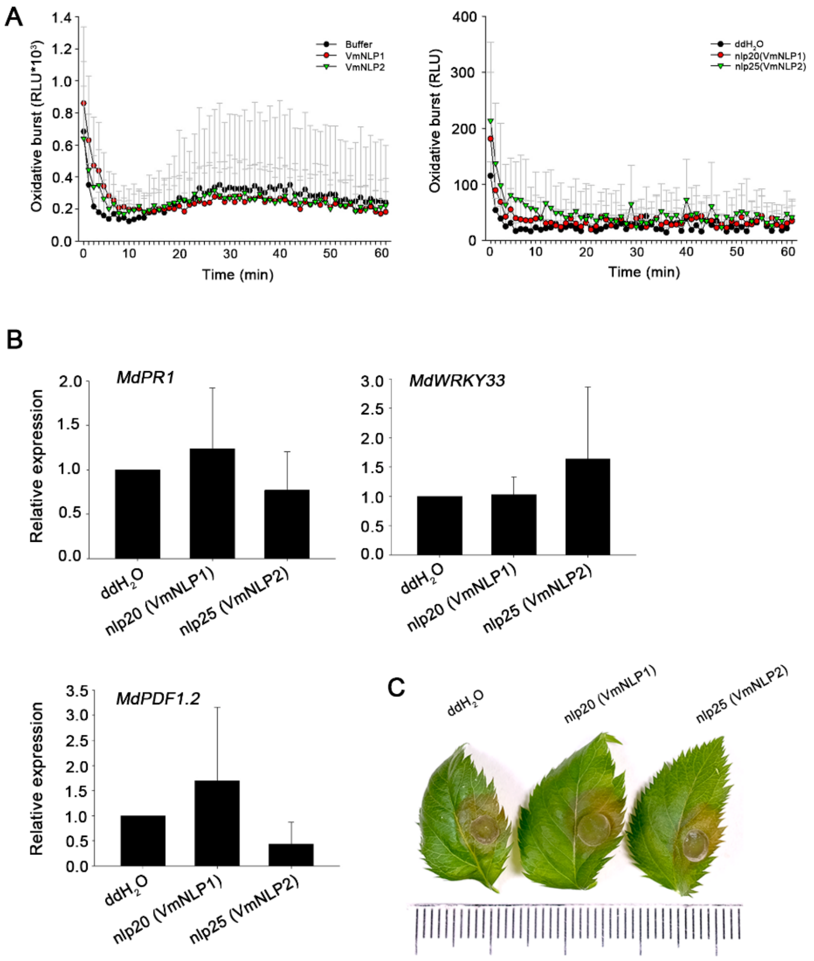

Supplementary Figure S9. The peptide nlp20 (VmNLP1) and nlp25 (VmNLP2) are unable to trigger immune responses in apple leaves.

(A) Oxidative burst in *Malus domestica* treated with 1 μM peptides (nlp20 (VmNLP1), nlp25 (VmNLP2)) or 1 μM purified recombination proteins (VmNLP1 and VmNLP2), with ddH2O and buffer as negative controls. Values are shown as relative light units (RLU). **(B)** nlp20 (VmNLP1) and nlp25 (VmNLP2) failed to activate transcript accumulation of defense-related genes in apple leaves. Micro-propagated apple rootstock plants were vacuum pressure-infiltrated with 1 μM peptides (nlp20 (VmNLP1) and nlp25 (VmNLP2)) or ddH2O control. Gene expression was assessed at 24 h post infiltration using qRT-PCR. *MdH* was used as an endogenous reference. Means and SEs were calculated from three independent biological replicates. (**C**) Symptom of pre-treated apple leaves 24 h post inoculation of *V. mali*. Apple leaves were vacuum pressure-infiltrated with 1 μM synthetic nlp20 (VmNLP1) and nlp25 (VmNLP2) peptides 24 h prior to inoculation of *V. mali*. ddH2O was used as a negative control. The experiments were performed three times with similar results.

Table S1 Primers used in this study

Primer	Sequence (5'-3')
**Gene deletion in *V. mali***	
VmNLP1-1F	AAGGGCTGTGGAGGGTTA
VmNLP1-2R	CAGATACGGCAGAGAAATCGCAACCTCAAAGGGCGGATGTGAAGA
VmNLP1-3F	GTTTAGATTCCAAGTGTCTACTGCTGGCTGGGCAGGTGTTTATGTG
VmNLP1-4R	AGCAGGTCAGGGTAGCAG
VmNLP1-5F	TCCGCTCACTTCTGTCTG
VmNLP1-6R	GTCCTTGGGGAAATACCT
VmNLP1-CF	TGGTCAACTTCAGCAACAA
VmNLP1-CR	AAGGCGAAGATAAGAGTGAT
VmNLP1-7F	CATCCCTGGAAATGGAGT
VmNLP1-GR	GGCGATACCGTAAAGCAC
VmNLP1-GF	AAGAACTTGGCGGCGAATG
VmNLP1-8R	AGGGGACCGATGCTACTGGA
G852-F	TCGGCTATGACTGGGCACAACA
G850-R	GAGCGGCGATACCGTAAAGCAC
VmNLP2-1F	AGTATCGCAACCAGTCCC
VmNLP2-2R	CAGATACGGCAGAGAAATCGCAACCTCCGAGCACCAAATAACCAT
VmNLP2-3F	GTTTAGATTCCAAGTGTCTACTGCTGGCGGGCATTTATGGTAGGAA
VmNLP2-4R	AGGGGATTCTCGTCAGTC
VmNLP2-5F	CCTCGCTCAACGCAAAAC
VmNLP2-6R	CTTACCGCCCCAGTCCTT
VmNLP2-CF	CGGAAACCACGTCCTTCA
VmNLP2-CR	GGGATTCTCGTCAGTCGC
NLP2-7F3	ATCCCGCTATGGTTATTTG
NLP2-GR3	AGCCAACGCTATGTCCTG
VmNLP2-GF	CTCCTGCCGAGAAAGTATCCA
VmNLP2-8R	TGCTTGTCATCCAACACCGT
NEO-F	GAGGTTGCGATTTCTCTGCCGTATCTG
NEO-R	GCCAGCAGTAGACACTTGGAATCTAAAC
**Gene complementation and overexpression in *V. mali***	
HB-PDL2-VmNLP1-F1	TCTCATCACCATCACCATCACAGGCAGCAAAGACAAATCAATC
HB-PDL2-VmNLP1-R1	GTAGCGGTGGCCGAGTCCGTCG
HB-PDL2-VmNLP1-F2	CGGACTCGGCCACCGCTACGACTGGGAGGGCGCCATCGT
HB-PDL2-VmNLP1-R2	TCGCCCTTGCTCACCCTCGACTAGAAGGTAGCTTTCGCCA
HB-PDL2-VmNLP2-F1	TCTCATCACCATCACCATCACCAATAGTATCGCAACCAGTCCC
HB-PDL2-VmNLP2-R1	ATGACGATGGCCGACACCGACG
HB-PDL2-VmNLP2-F2	CGGTGTCGGCCATCGTCATGATTGGGAGAATGCCGTTAT
HB-PDL2-VmNLP2-R2	TCGCCCTTGCTCACCCTCGATTACGACAGATCCAGATATT
OE-PDL2-VmNLP1-XhoI-F	TCTCATCACCATCACCATCACATGGCCGTCCTCCGCTCACT
OE-PDL2-VmNLP1-XhoI-R	TCGCCCTTGCTCACCCTCGACTAGAAGGTAGCTTTCGCCA

OE-PDL2-VmNLP2-XhoI-F	TCTCATCACCATCACCATCACATGTTTTCAAAACTCACAGT
OE-PDL2-VmNLP2-XhoI-R	TCGCCCTTGCTCACCCTCGATTACGACAGATCCAGATATT

Transient expression in *N. benthamiana*

pCAMBIA1300-VmNLP1-HA-F	CGGGGGACGAGCTCGGTACCATGGCCGTCCTCCGCTCAC
pCAMBIA1300-VmNLP1-HA-R	ACGTCGTATGGGTAGGTACCGAAGGTAGCTTTCGCCAAG
pCAMBIA1300-VmNLP2-HA-F	CGGGGGACGAGCTCGGTACCATGTTTTCAAAACTCACAG
pCAMBIA1300-VmNLP2-HA-R	ACGTCGTATGGGTAGGTACCCGACAGATCCAGATATTC
pCAMBIA1300-NSVmNLP2-HA-F	CGGGGGACGAGCTCGGTACCATGGCGCCCTCGAGGACCCACT
pCAMBIA1300-NSVmNLP2-HA-R	ACGTCGTATGGGTAGGTACCCGACAGATCCAGATATTC
pCAMBIA1300-GFP-HA-F	CGGGGGACGAGCTCGGTACCATGGTGAGCAAGGGCGAGGA
pCAMBIA1300-GFP-HA-R	ACGTCGTATGGGTAGGTACCCTACTTGTACAGCTCGTCCA
pICH86988-INF1-mCherry-F	CATTTACAATTATCGATATGACATCTCTCCATACGT
pICH86988-INF1-mCherry-R	CTCACCCTAGGACTAGTTAGCGACGCACACGTAGAC
pCAMBIA1300-VmNLP1-H127Y-HA-F1	CGGGGGACGAGCTCGGTACCATGGCCGTCCTCCGCTCAC
pCAMBIA1300-VmNLP1-H127Y-HA-R1	GTAGCGGTGGCCGAGTCCGTCG
pCAMBIA1300-VmNLP1-H127Y-HA-F2	CGGACTCGGCCACCGCTACGACTGGGAGGGCGCCATCGT
pCAMBIA1300-VmNLP1-H127Y-HA-R2	ACGTCGTATGGGTAGGTACCGAAGGTAGCTTTCGCCAAG
pCAMBIA1300-VmNLP2-Y137H-HA-F1	CGGGGGACGAGCTCGGTACCATGTTTTCAAAACTCACAGT
pCAMBIA1300-VmNLP2-Y137H-HA-R1	ATGACGATGGCCGACACCGACG
pCAMBIA1300-VmNLP2-Y137H-HA-F2	CGGTGTCGGCCATCGTCATGATTGGGAGAATGCCGTTAT
pCAMBIA1300-VmNLP2-Y137H-HA-R2	ACGTCGTATGGGTAGGTACCCGACAGATCCAGATATTC

qRT-PCR analysis

qRT-VmNLP1-F	CTACAGGTGCATCCAACGGAG
qRT-VmNLP1-R	CCTTGGGGAAATACCACGAAT
qRT-VmNLP2-F	TGCTGAAGTGGCAACCATACC
qRT-VmNLP2-R	GCCTGTAGAGCTGGAGCAGTCT
G6PDH-F	TCAGAACAAGTTCGAGGGCGACAA
G6PDH-R	TGAGGGCAATAGAGGGCTTGTTCA
qRT-AtActin2-F	CACTTGCACCAAGCAGCATGAAGA
qRT-AtActin2-R	AATGGAACCACCGATCCAGACACT
qRT-AtPR1-F	GCTAACTACAACTACGCTGCGAA
qRT-AtPR1-R	TCTCGTTCACATAATTCCCACGA
qRT-AtWRKY33-F	GAAACAAATGGTGGGAATGG
qRT-AtWRKY33-R	TGTCGTGTGATGCTCTCTCC
qRT-AtFRK1-F	GCCAACGGAGACATTAGAG
qRT-AtFRK1-R	CCATAACGACCTGACTCATC
qRT-MdH-F	CGTGATTGGGTACTTGGAAC
qRT-MdH-R	TGGCAAGTGACTGGGAATGA
qRT-MdPR1-F	GGCTCAGTCCTTATCCAATCCTC
qRT-MdPR1-R	GCCTGCTACTTTGTCATCCCACG
qRT-MdWRKY33-F	CGAAATTGTTCTCCGATTTC
qRT-MdWRKY33-R	GAATGGTTCTTCCACTGAAACC

qRT-MdPDF1.2-F	GTGTTGAGGCAAAGTCCAAATC
qRT-MdPDF1.2-R	CAGGTGTTTGCACAGTTGTTAG
qRT-NbEF1α-F	AAGGTCCAGTATGCCTGGGTGCTTGAC
qRT-NbEF1α-R	AAGAATTCACAGGGACAGTTCCAATACCA
qRT-NbPR1-F	CCGCCTTCCCTCAACTCAAC
qRT-NbPR1-R	GCACAACCAAGACGTACTGAG
qRT-NbPR2-F	AGGTGTTTGCTATGGAATGC
qRT-NbPR2-R	TCTGTACCCACCATCTTGC
qRT-NbPR4-F	GGCCAAGATTCCTGTGGTAGAT
qRT-NbPR4-R	CACTGTTGTTTGAGTTCCTGTTCCT

**Protein expression in *E. coli***	
pET28a-VmNLP2-F	GTGCCGCGCGGCAGCCATATGGCGCCCTCGAGGACCCACTC
pET28a-VmNLP2-R	ACGGAGCTCGAATTCGGATCCTTACGACAGATCCAGATATT

Supplementary Table S2. Specification of VmNLP1 and VmNLP2.

ID	GenBank	E Value	Length	Mw (kDa)	Signal peptide	Conversed cystines	Type
VmNLP1	KUI72711.1	$3E^{-38}$	238	24.96	1-19	2 (62,88)	1
VmNLP1	KUI72946.1	$2E^{-45}$	255	27.29	1-17	2 (67,93)	1

Original gel figures in this work

(refers to 1B) (refers to 6B)

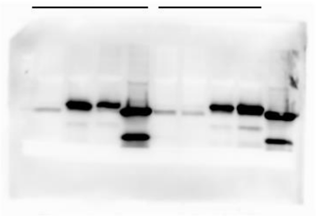

(refers to 1B)

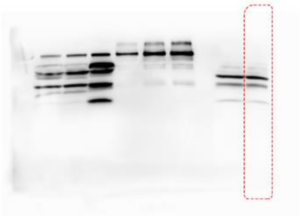

(refers to 2A)

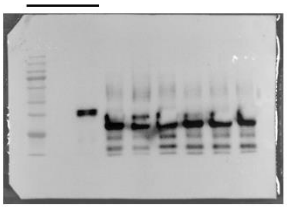

(refers to 1B) (refers to 6B)

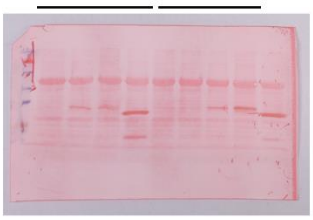

(refers to 1B)

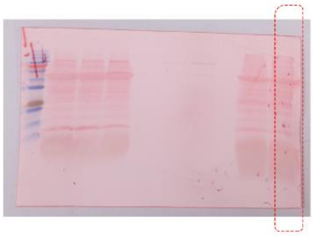

(refers to 2A)

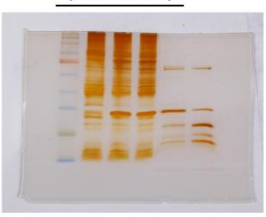

(refers to S2A)

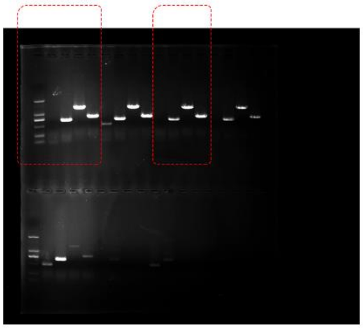

(refers to S2A)

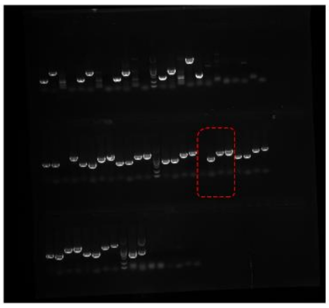

(refers to S2B)

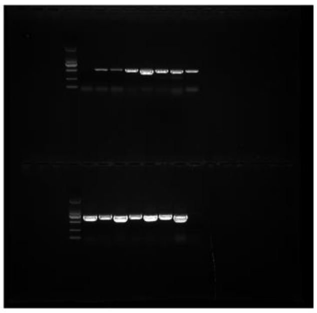

References

1. Böhm, H.; Albert, I.; Oome, S.; Raaymakers, T.M.; Van den Ackerveken, G.; Nürnberger, T. A conserved peptide pattern from a widespread microbial virulence factor triggers pattern-induced immunity in *Arabidopsis*. *PLoS Pathog* **2014**, *10*, e1004491.
2. Bailey, B.A.; Jennings, J.C.; Anderson, J.D. The 24-kDa protein from *Fusarium oxysporum* f. sp. *erythroxyli*: occurrence in related fungi and the effect of growth medium on its production. *Can. J. Microbiol.* **1997**, *43*, 45–55.
3. Dallal Bashi, Z.; Hegedus, D.D.; Buchwaldt, L.; Rimmer, S.R.; Borhan, M.H. Expression and regulation of *Sclerotinia sclerotiorum* necrosis and ethylene-inducing peptides (NEPs). *Mol. Plant Pathol.* **2010**, *11*, 43–53.
4. Santhanam, P.; van Esse, H.P.; Albert, I.; Faino, L.; Nürnberger, T.; Thomma, B.P. Evidence for functional diversification within a fungal NEP1-like protein family. *Mol. Plant-Microbe Interact.* **2013**, *26*, 278–286.
5. Schouten, A.; Van Baarlen, P.; Van Kan, J.A. Phytotoxic Nep1-like proteins from the necrotrophic fungus *Botrytis cinerea* associate with membranes and the nucleus of plant cells. *New Phytol.* **2008**, *177*, 493–505.
6. Garcia, O.; Macedo, J.A.; Tibúrcio, R.; Zaparoli, G.; Rincones, J.; Bittencourt, L.M.; Ceita, G.O.; Micheli, F.; Gesteira, A.; Mariano, A.C. Characterization of necrosis and ethylene-inducing proteins (NEP) in the basidiomycete *Moniliophthora perniciosa*, the causal agent of witches' broom in *Theobroma cacao*. *Mycol. Res.* **2007**, *111*, 443–455.
7. Cabral, A.; Oome, S.; Sander, N.; Küfner, I.; Nürnberger, T.; Van den Ackerveken, G. Nontoxic Nep1-like proteins of the downy mildew pathogen *Hyaloperonospora arabidopsidis*: repression of necrosis-inducing activity by a surface-exposed region. *Mol. Plant-Microbe Interact.* **2012**, *25*, 697–708.
8. Kanneganti, T.-D.; Huitema, E.; Cakir, C.; Kamoun, S. Synergistic interactions of the plant cell death pathways induced by *Phytophthora infestans* Nep1-like protein PiNPP1. 1 and INF1 elicitin. *Mol. Plant-Microbe Interact.* **2006**, *19*, 854–863.
9. Qutob, D.; Kamoun, S.; Gijzen, M. Expression of a *Phytophthora sojae* necrosis-inducing protein occurs during transition from biotrophy to necrotrophy. *Plant J.* **2002**, *32*, 361–373.
10. Veit, S.; Wörle, J.M.; Nurnberger, T.; Koch, W.; Seitz, H.U. A novel protein elicitor (PaNie) from *Pythium aphanidermatum* induces multiple defense responses in carrot, *Arabidopsis*, and tobacco. *Plant Physiol.* **2001**, *127*, 832–841.
11. Mattinen, L.; Tshuikina, M.; Mäe, A.; Pirhonen, M. Identification and characterization of Nip, necrosis-inducing virulence protein of *Erwinia carotovora* subsp. *carotovora*. *Mol. Plant-Microbe Interact.* **2004**, *17,* 1366–1375.
12. Ottmann, C.; Luberacki, B.; Küfner, I.; Koch, W.; Brunner, F.; Weyand, M.; Mattinen, L.; Pirhonen, M.; Anderluh, G.; Seitz, H.U. A common toxin fold mediates microbial attack and plant defense. *Proc. Natl. Acad. Sci. USA* **2009,** 106, (25), 10359-10364.
