# Supplementary figures and images for: Nep1-like Proteins from Valsa mali Differentially Regulate Pathogen Virulence and Response to Abiotic Stresses
Source: J Fungi (Basel). 2021 Oct 4;7(10):830. doi: 10.3390/jof7100830 (PMC8539816; doi:10.3390/jof7100830)

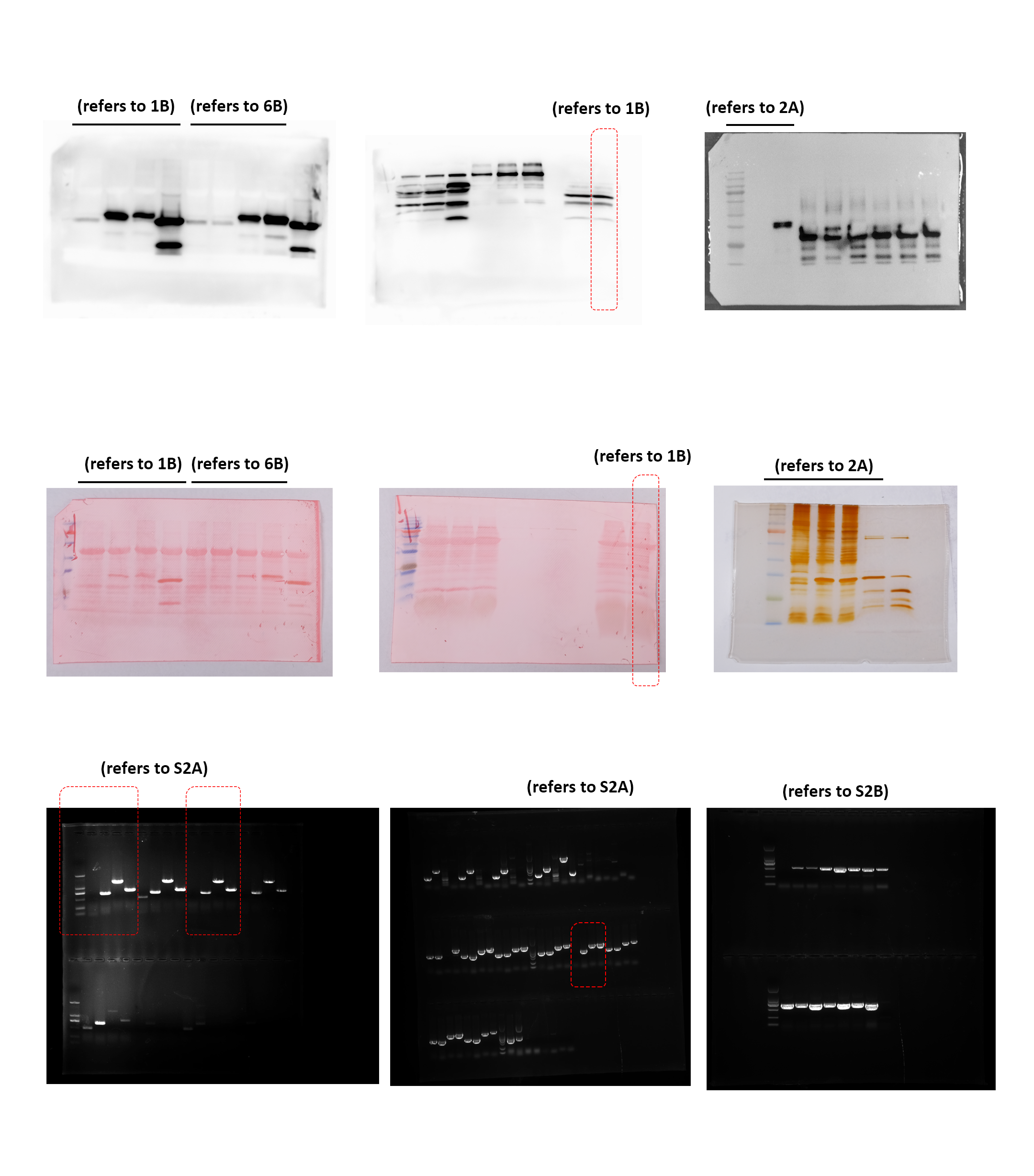

Supplement: Supplementary file 1 [file jof-07-00830-s001.zip › Original gel data.tif]

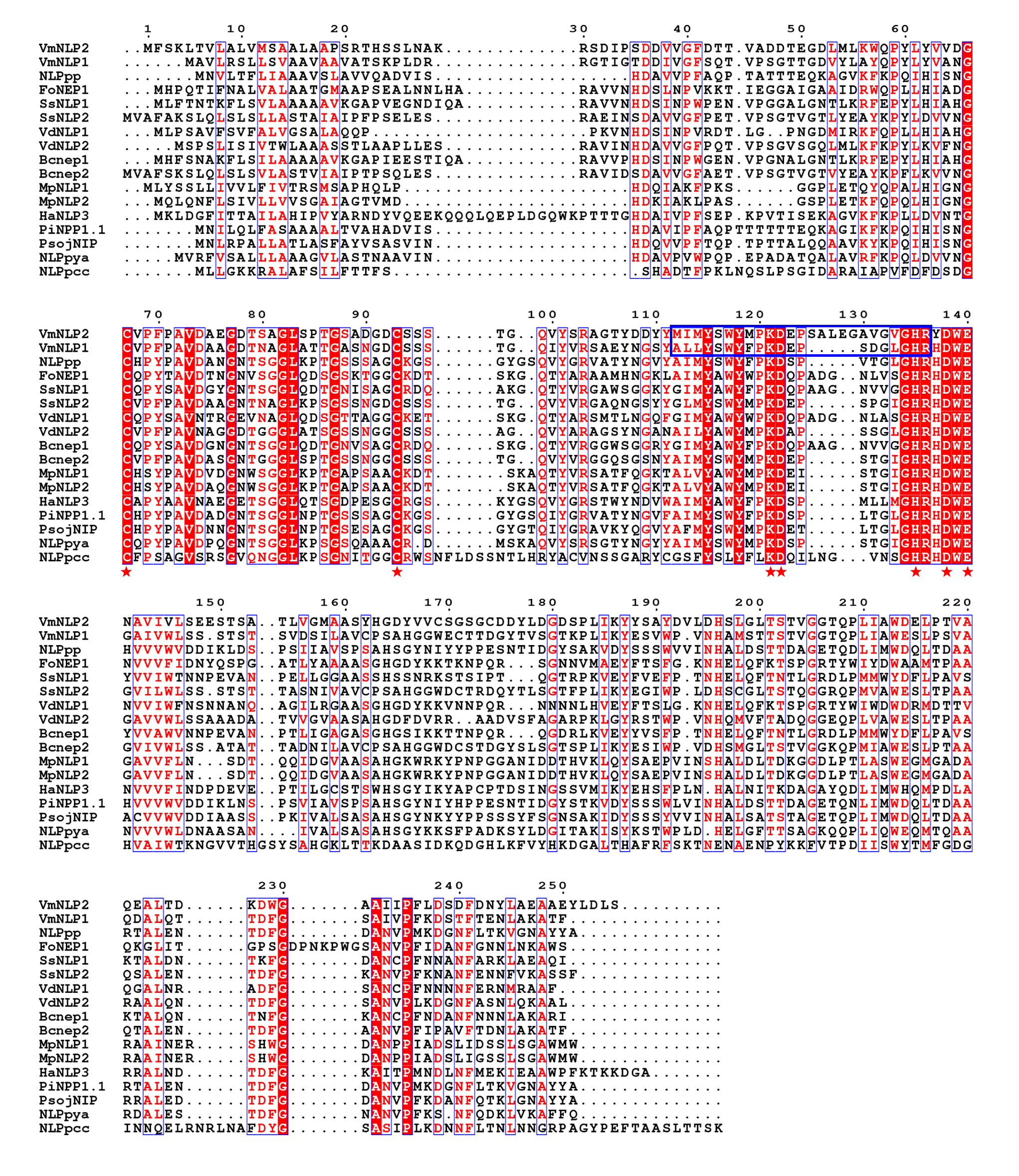

Supplement: Supplementary file 1 [file jof-07-00830-s001.zip › Supplementary Figure S1.tif]

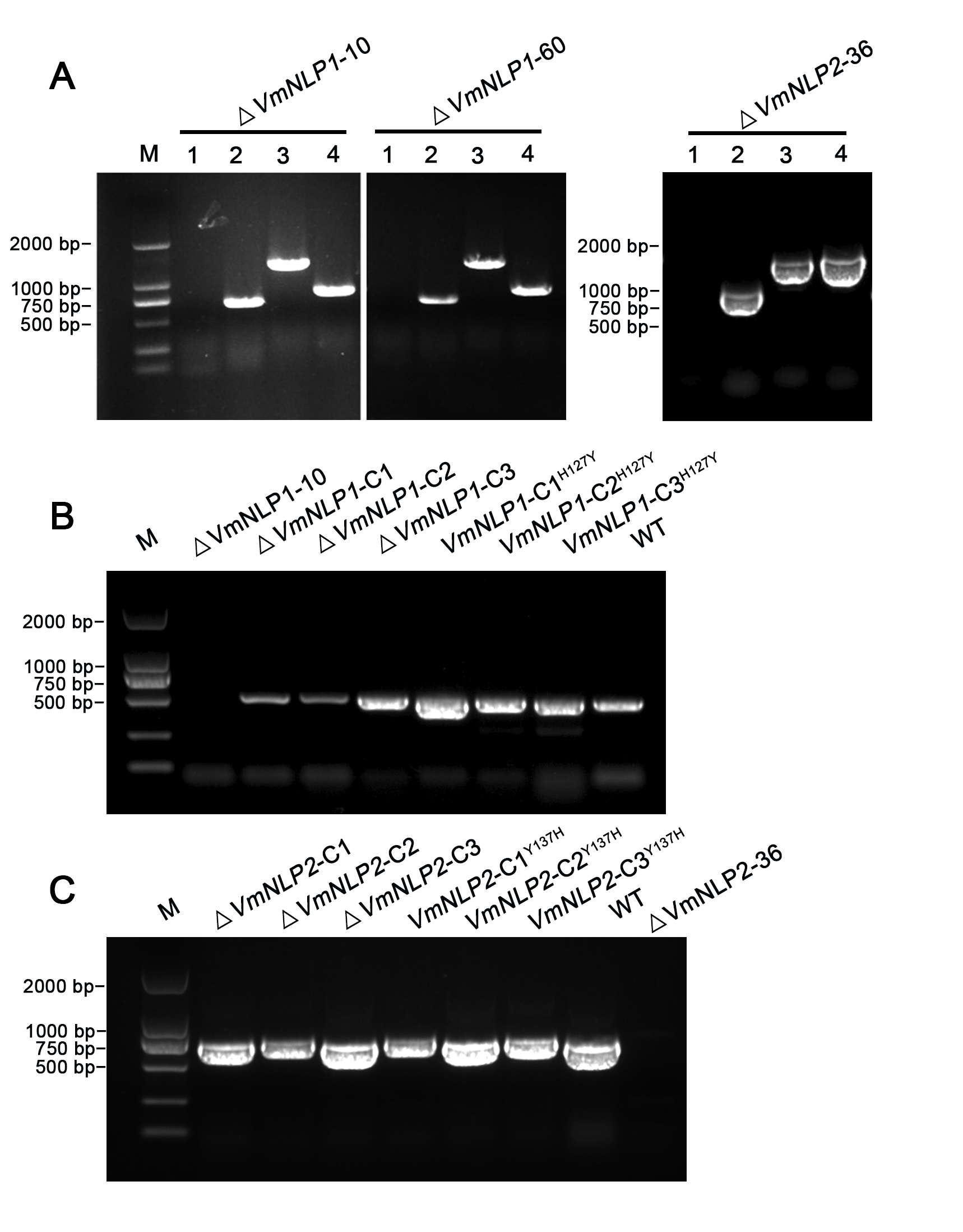

Supplement: Supplementary file 1 [file jof-07-00830-s001.zip › Supplementary Figure S2.tif]

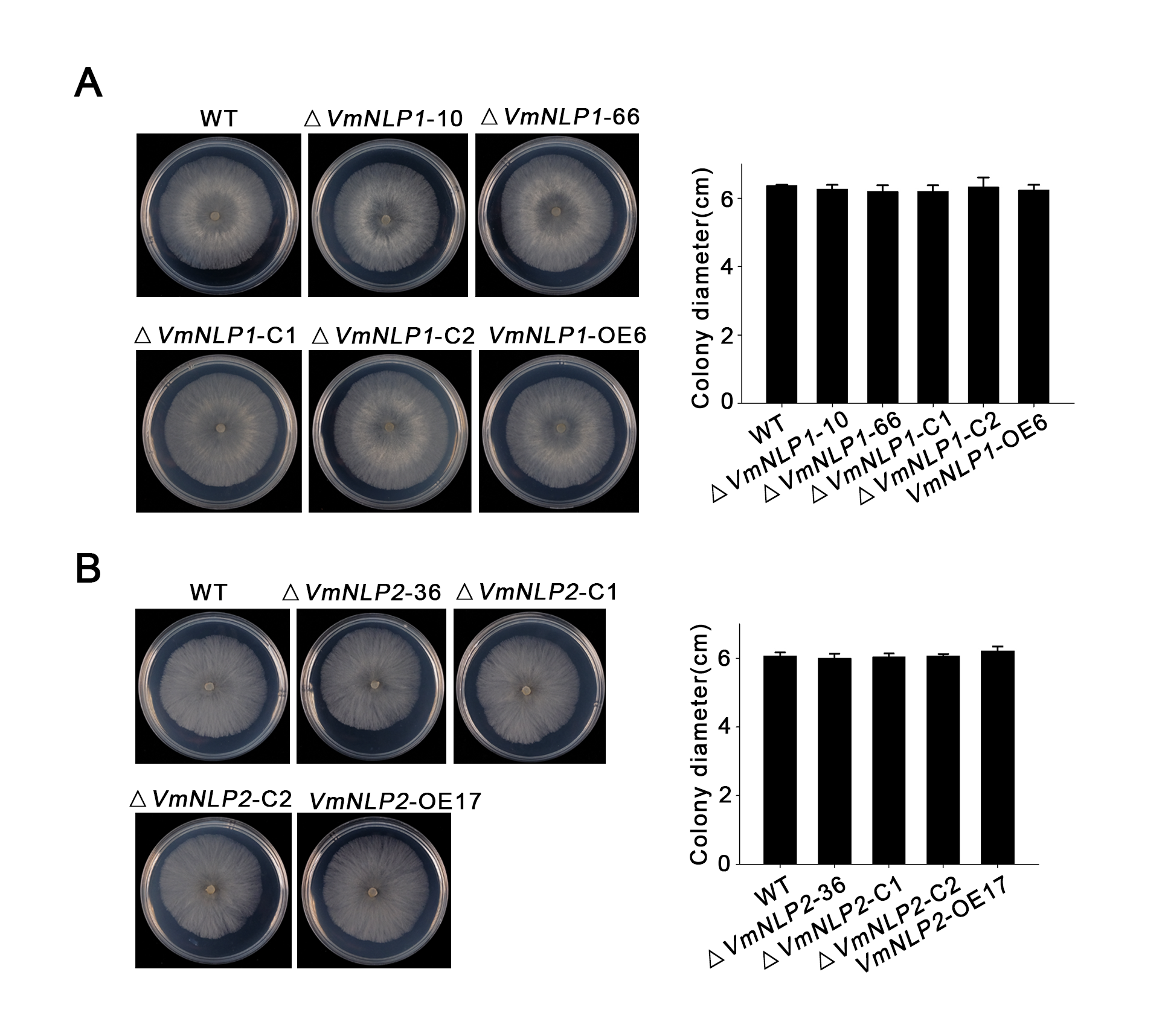

Supplement: Supplementary file 1 [file jof-07-00830-s001.zip › Supplementary Figure S3.tif]

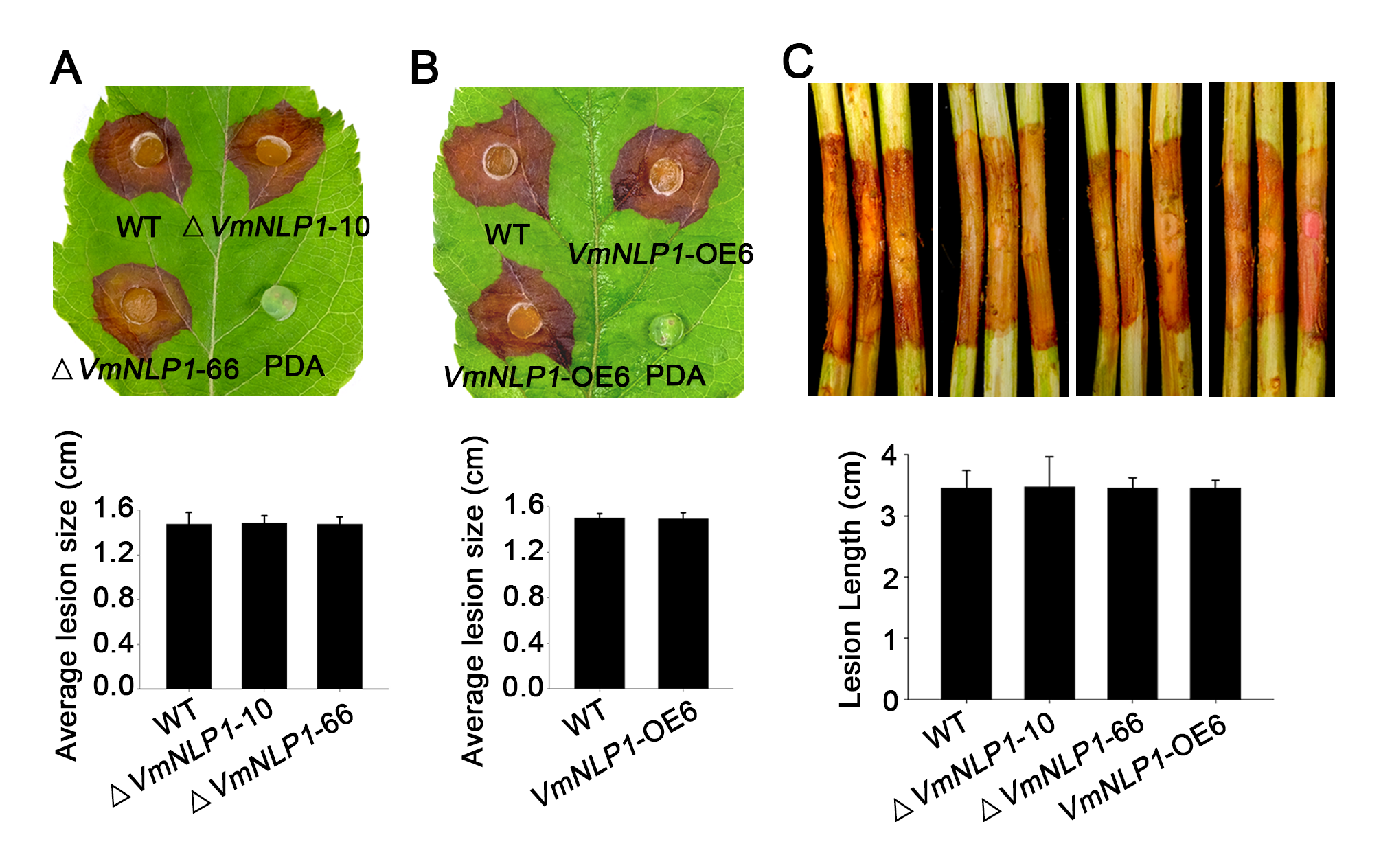

Supplement: Supplementary file 1 [file jof-07-00830-s001.zip › Supplementary Figure S4.tif]

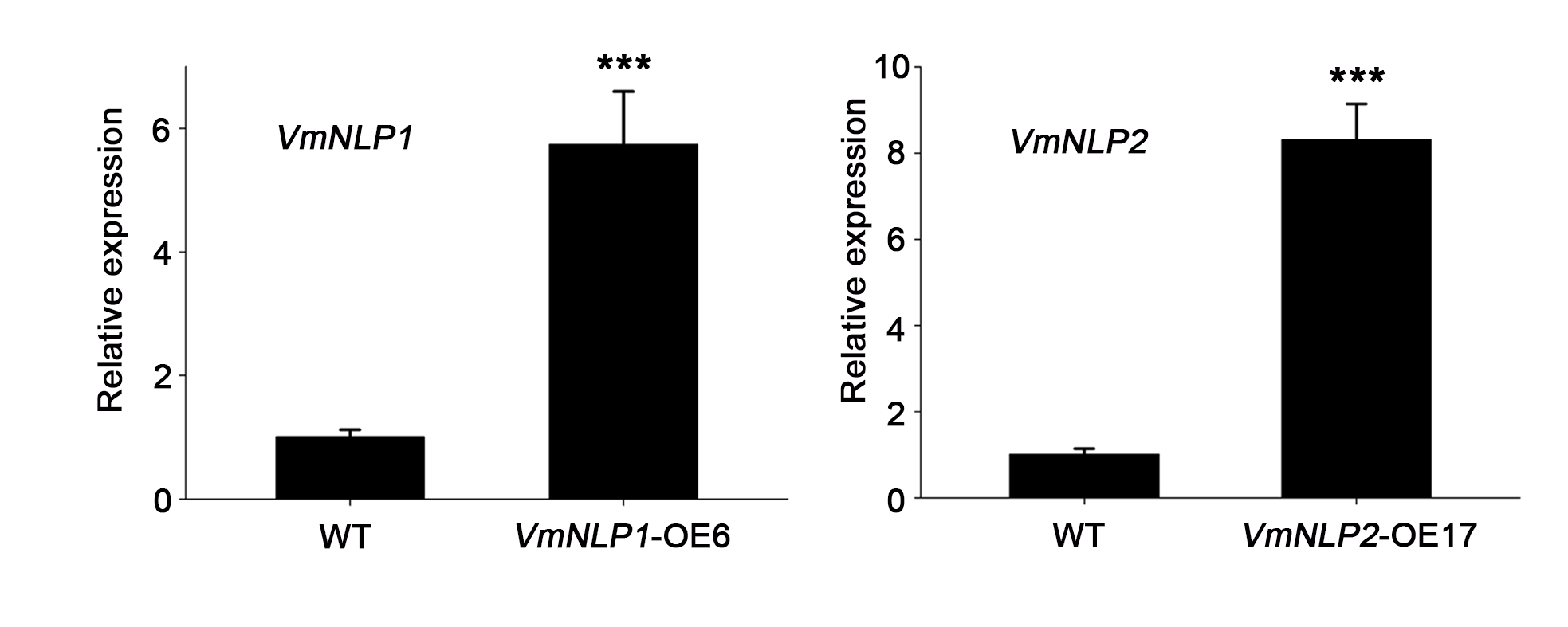

Supplement: Supplementary file 1 [file jof-07-00830-s001.zip › Supplementary Figure S5.tif]

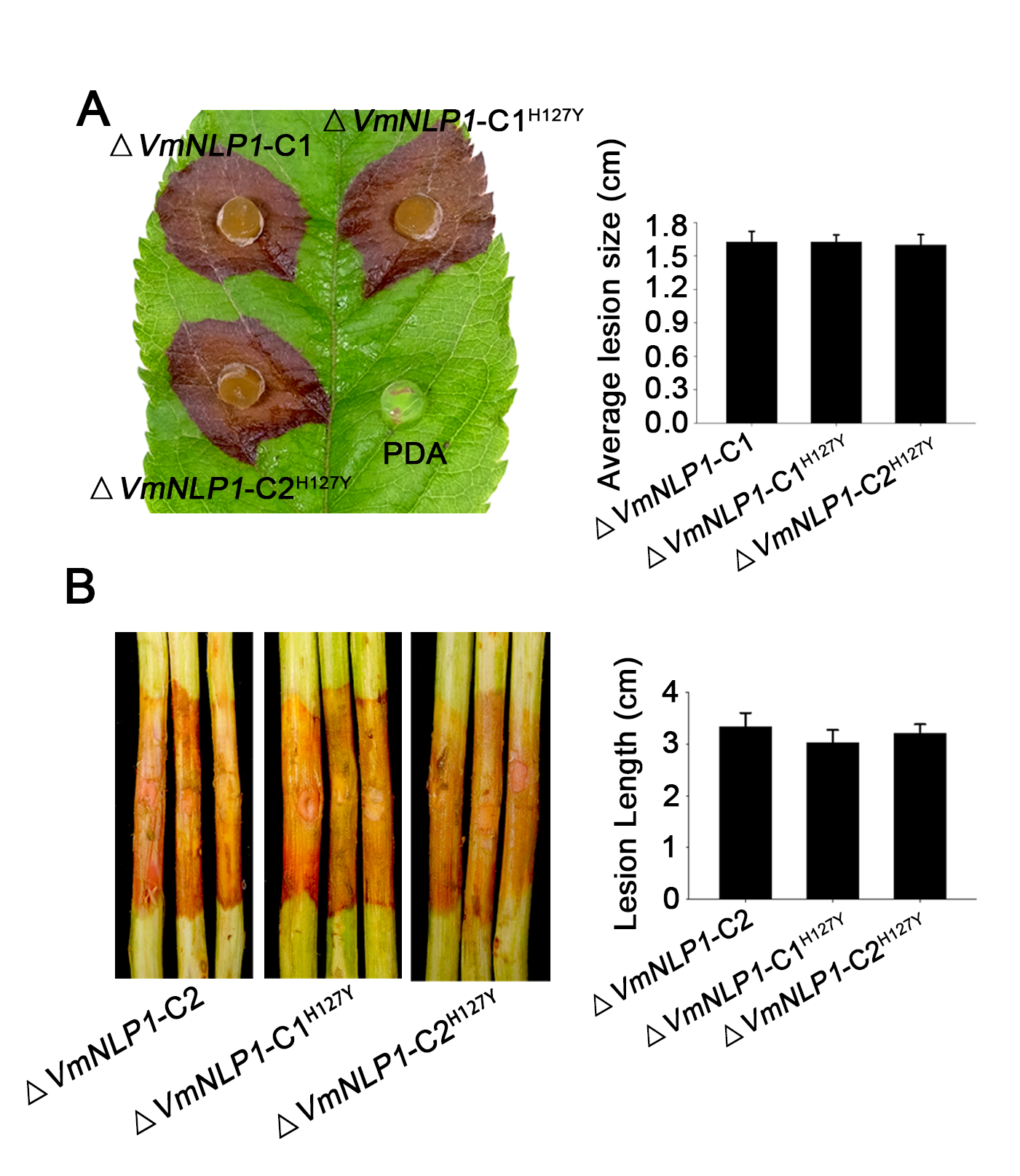

Supplement: Supplementary file 1 [file jof-07-00830-s001.zip › Supplementary Figure S6.tif]

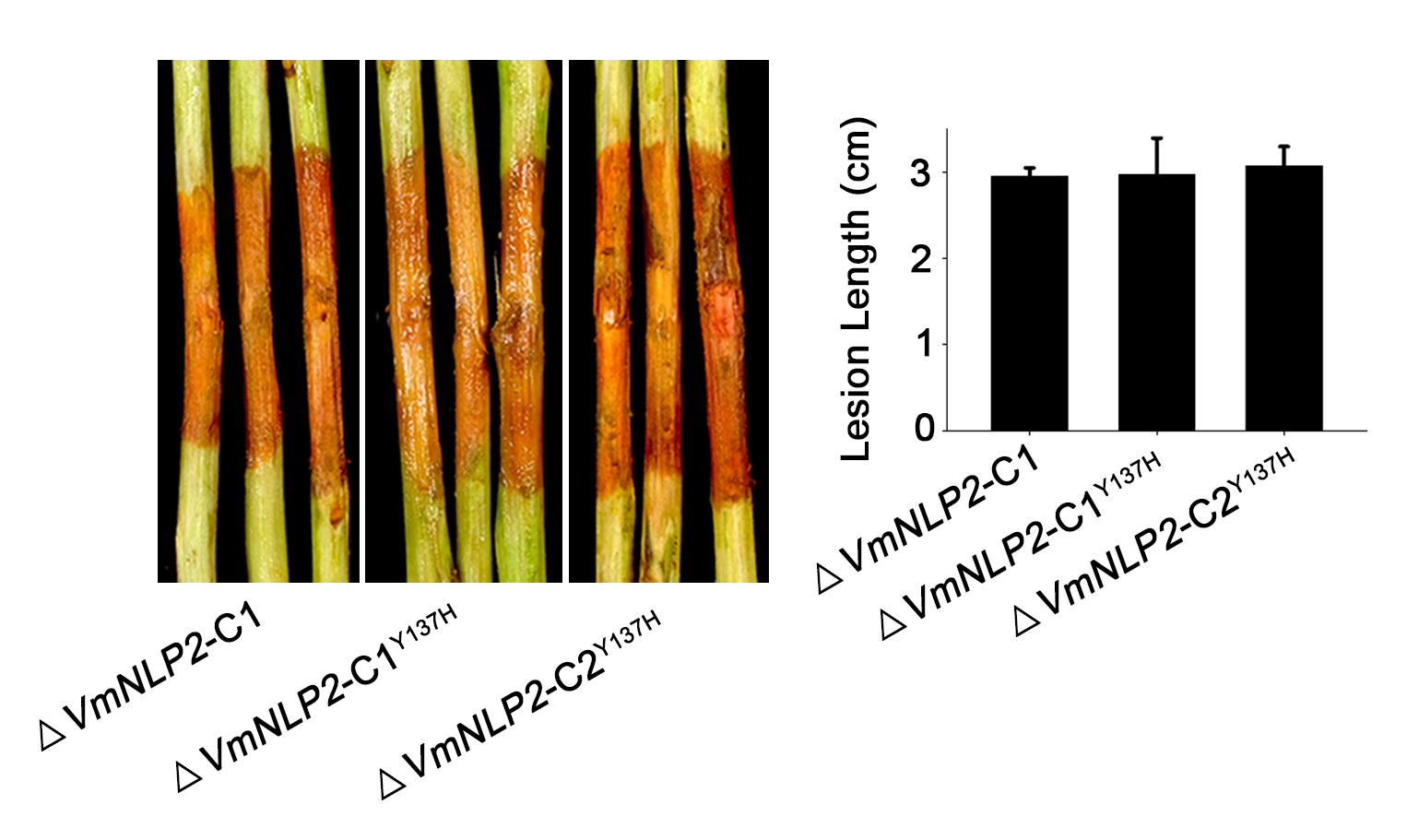

Supplement: Supplementary file 1 [file jof-07-00830-s001.zip › Supplementary Figure S7.tif]

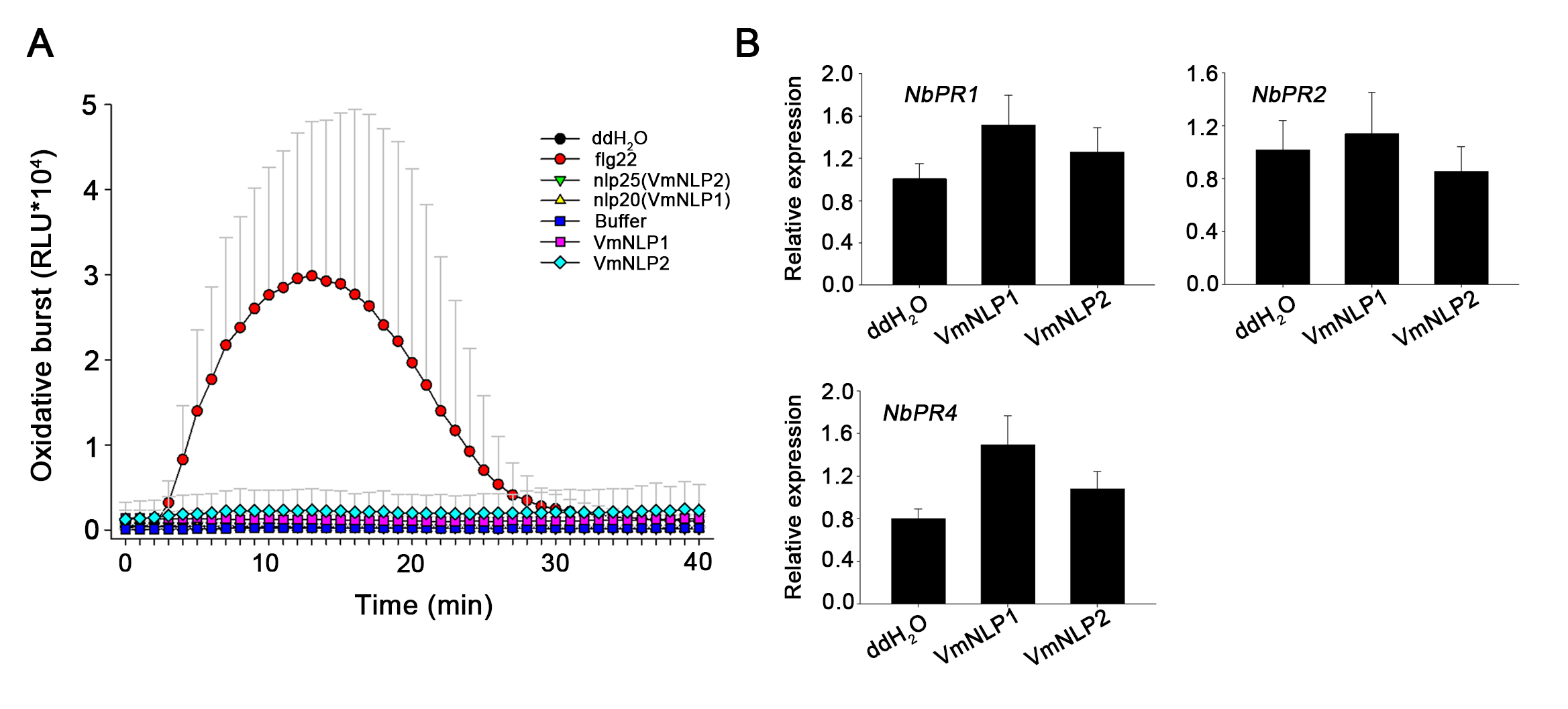

Supplement: Supplementary file 1 [file jof-07-00830-s001.zip › Supplementary Figure S8.tif]

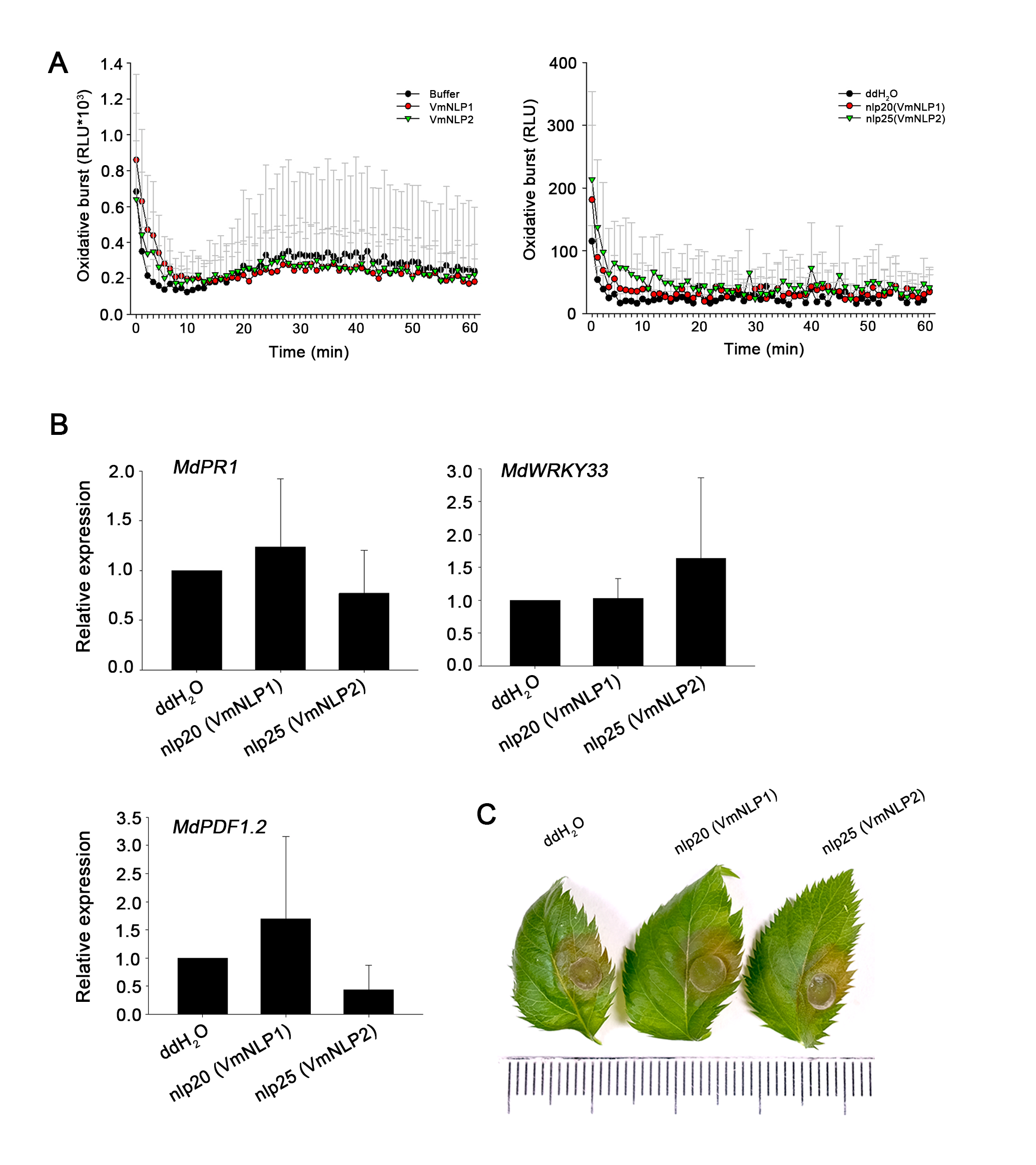

Supplement: Supplementary file 1 [file jof-07-00830-s001.zip › Supplementary Figure S9.tif]
